# Successful Management, in a Low-Resource Setting, of Disseminated Tuberculosis in a 3-Year Old Boy: A Case Report

**DOI:** 10.3390/pathogens12091163

**Published:** 2023-09-15

**Authors:** Josina Chilundo, Arlindo Muhelo, Zita Ahivaldino, Helton Zucula, Sheila Macuácua, Ana Cristina Mussagi, Damiano Pizzol, Lee Smith, Giuseppe Maggioni

**Affiliations:** 1 Department of Pneumology, Central Hospital of Maputo, Maputo 1113, Mozambique; jchalufo@yahoo.com.br; 2 Faculty of Medicine, Eduardo Mondlane University Maputo, Maputo 1113, Mozambique; 3 Department of Paediatry, Central Hospital of Maputo, Maputo 1113, Mozambique; arlindomuhelo@gmail.com (A.M.); ahyvaldo@hotmail.com (Z.A.); heltonzucula@gmail.com (H.Z.); sheyla.macuacua@gmail.com (S.M.); hannahcristinna89@gmail.com (A.C.M.); 4 Operative Research Unit, Doctors with Africa Cuamm, Beira 1100, Mozambique; 5 Centre for Health, Performance and Wellbeing, Anglia Ruskin University, Cambridge CB1 1PT, UK; lee.smith@aru.ac.uk; 6 Department of Medicine, University of Padua, 35128 Padova, Italy; maggioni.giuseppe@hotmail.it

**Keywords:** tuberculosis, disseminated TB, low-income setting

## Abstract

Disseminated or military tuberculosis (TB) is defined as the presence of at least two non-contiguous sites of *Mycobacterium tuberculosis*, occurring as a result of progressive primary infection, reactivation and spread of a latent focus or due to iatrogenic origin. Disseminated TB represents a life-threatening condition, especially in at-risk children and when diagnosis and treatment are delayed. We report on a case of a 3-year old boy who presented with long-lasting unrecognised disseminated TB that was successfully managed in a low-resource setting.

## 1. Introduction

Tuberculosis (TB) is among the top ten causes of death worldwide. TB represents a marker of inequality as the overwhelming burden of TB is found among low- and middle-income countries, where it is estimated that over 90% of global TB cases and deaths occur [1,2]. Disseminated or miliary tuberculosis is defined as the presence of at least two non-contiguous sites resulting from lymphohematogenous dissemination of *Mycobacterium tuberculosis*, occurring as a result of progressive primary infection, reactivation and spread of a latent focus or due to iatrogenic origin [3]. Disseminated TB represents a life-threatening condition, especially in at-risk children and when diagnosis and treatment are delayed [4,5,6]. Clinical presentation of disseminated TB is nonspecific, it is commonly associated with fever of unknown origin and, depending on involved sites, with other generic symptoms attributable to other common diseases [7]. In addition, in low-income settings, the paucity of tools available for confirmatory laboratory diagnosis, such as the low sensitivity of acid-fast bacilli (AFB) smear, time-consuming cultures, and the inability to easily detect miliary changes in a chest X-ray, makes diagnosis a very challenging task [7]. The time from symptoms presentation to diagnosis is highly variable, ranging from few days to several months depending on health professionals’ training, diagnostic tools, and clinical presentation that usually includes subacute or chronic constitutional symptoms such as fever, weight loss, and night sweats. Commonly, most children are treated based on a combination of clinical and radiologic signs suggestive of TB without bacteriological confirmation [8]. To date, mortality due to disseminated TB is still high, ranging from 25% to 30% mainly due to the delay in diagnosing and the onset of meningismus, liver cirrhosis, leukopenia, leukocytosis, advancing age, presence of underlying disease, altered mental status, and night sweats [9]. In the present paper, a case of a 3-year-old boy who presented with long-lasting unrecognised disseminated TB that was successfully managed in a low-resource setting, is reported.

## 2. Case Report

**Figure 1:**
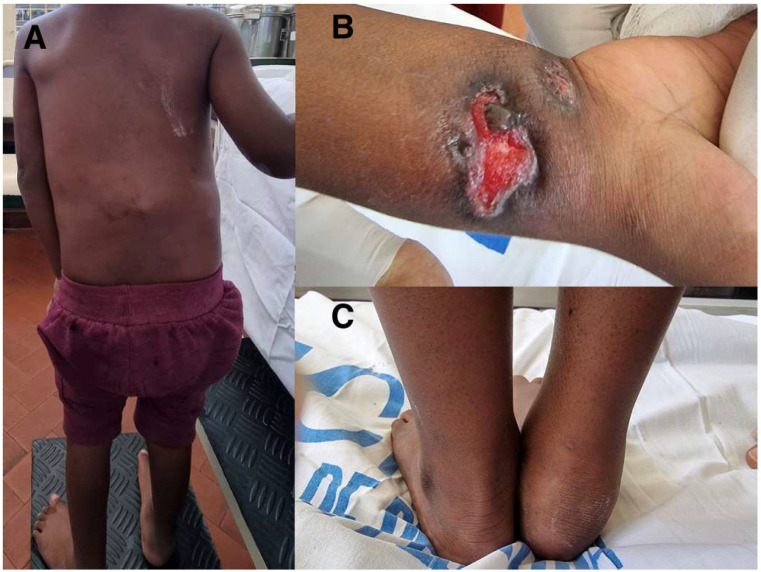
Disseminated tuberculosis at presentation in 3-year-old boy: deformity of the lumbar spine (**A**), solution of skin continuity on the distal right forearm (**B**), and cold, soft oedema in the right ankle (**C**).

A 3-year-old boy was transferred, on June 2023, from a rural health centre to a regional hospital in Mozambique due to suspected acute flaccid paralysis and paraparesis. Eight months before, October 2022, he developed intermittent fever, which improved with paracetamol administration. In December 2022 the clinical status evolved with the appearance of a swelling in the right wrist, treated with an incision and drainage and prescription of paracetamol and antibiotics in a rural health centre. After a week, further swelling appeared in the region of the spine along with an oedema in the right ankle, accompanied by intense and progressive pain, and paracetamol was prescribed in the same health centre. In April 2023, the condition worsened with increased swelling in the spine region, worsening pain, difficulty in sitting and walking. He also mentioned occasional dry cough, night sweats, asthenia, and weight loss, denying chest pain and dyspnea. Due to the worsening conditions, the health centre decided to transfer the boy to a regional hospital, but due to lack of means, the mother was recommended to wait at home with the boy for the availability of transport. After two more months and the further worsening of the condition he was transferred to the regional hospital on suspicion of acute flaccid paralysis and paraparesis. He presented with a deformity of the lumbar spine (Figure 1A), solution of skin continuity on the anterior surface of the distal 1/3 of the right forearm, measuring approximately 2.5 × 1.5 cm (Figure 1B), and a cold, soft oedema in the region of the right ankle (Figure 1C). He also presented a pathological gait (Alderman), with a tendency to support the left arm over the waist. The initial diagnosis was Pott’s Disease, and he started the specific following treatment on daily regimen: isoniazid (H), rifampicin (R), ethambutol (E), and pyrazinamide (Z) for 2 months followed by HRE for 10 months. The curettage was performed on the wound that was subsequently cleaned and medicated daily. Five days after starting treatment, he developed an episode of generalised tonic-clonic seizures lasting two minutes without sphincter relaxation, associated with fever. The results of the biochemical test performed at admission, after 2 and 5 days, are reported in Table 1.

**Figure 2:**
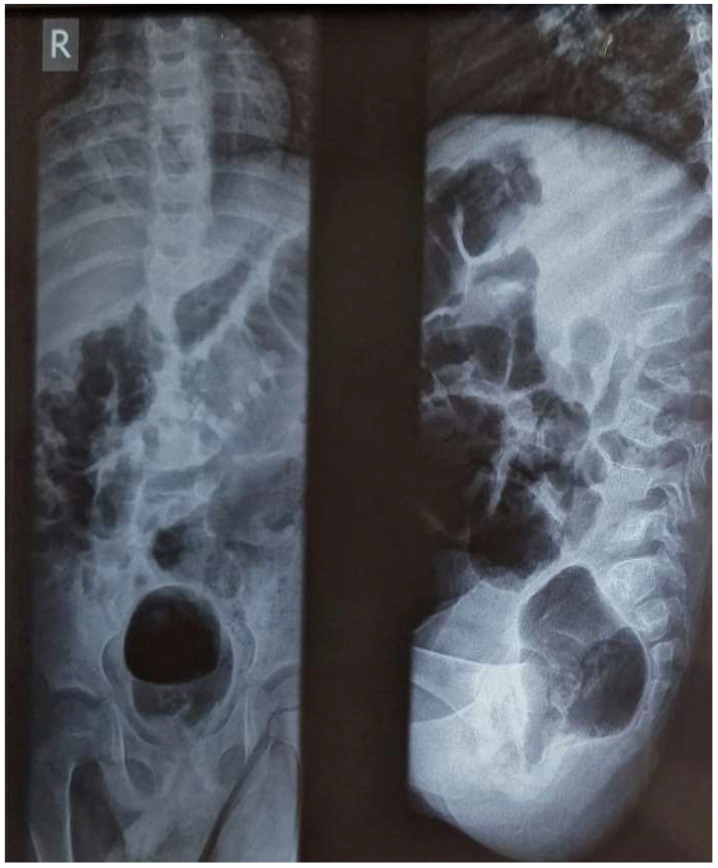
Plain X-ray of the spine.

**Figure 3:**
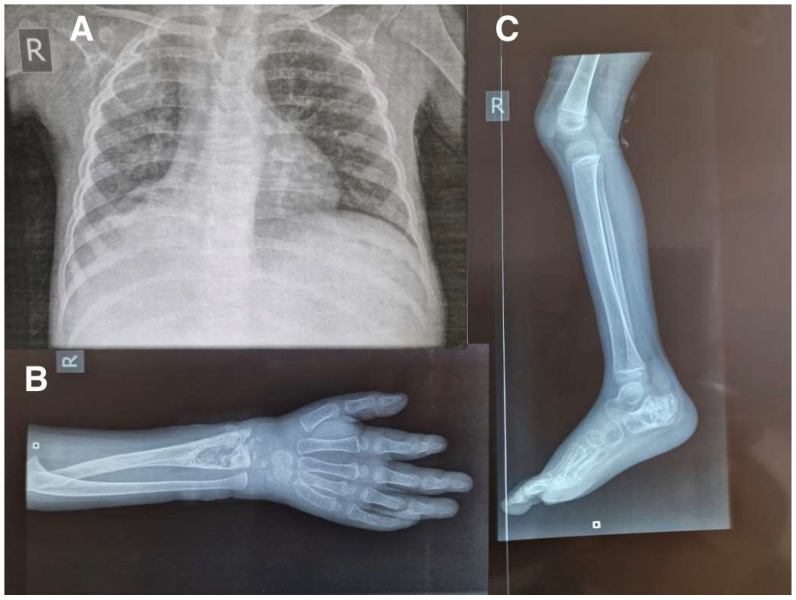
The chest x-ray (**A**), X-ray of the right forearm (**B**) and the X-ray of the right leg (**C**).

In addition to those tests at admission, HIV and GeneXpert MTB/RIF test results were negative. A plain X-ray of the spine showed a loss of anterior and posterior alignment of L2 and L3, change in height of the L2 vertebral body and altered bone density of the L2 and L3 vertebral bodies (Figure 2). The chest X-ray showed heterogeneous infiltration in both lung fields (Figure 3A) while the X-ray of the right forearm showed an osteolytic lesion in the distal 1/3 of the radius (Figure 3B), and the X-ray of the right leg showed signs of calcaneal osteolysis (Figure 3C). Based on clinical history, examination, and X-ray results, the diagnosis of disseminated TB, including Pott’s Disease pulmonary and cutaneous TB, was made. The conditions improved slowly but consistently, with gait improvement healing of the forearm injury and reduction of ankle oedema. The boy was discharged after 23 days with a one-year prescribed treatment and follow-up in 12 months if no complications occur.

## 3. Discussion

Mozambique has one of the highest TB burdens in the world, with an estimated TB incidence rate of 551/100,000 population in 2015. Moreover, TB treatment covers only 38% of the population, and an increasing rate of TB–HIV co-infection is usually documented (36% of TB patients were HIV-positive) [10,11]. Despite these dramatic data, TB and especially disseminated TB remain underrecognized and undiagnosed due to a lack of well-trained health workers and adequate tools for assessment and appropriate follow-up. In particular, in the present case, eight months elapsed from the first visit to the correct diagnosis. The reasons for such a delay are multiple. First, the child had no specific TB symptoms and health workers were not able to identify the disease, then the rural health centre was not properly equipped for deeper investigation. Finally, two additional months were necessary to wait to transfer the child. As precise and timely diagnosis is crucial for a favourable prognosis. It is thus essential to, on the one hand, to strengthen existing structures and, on the other, to find alternative solutions. In this regard, recent evidence suggests the possible role of Stool Xpert MTB/RIF on stool samples, especially in sub-Saharan settings, although its diagnostic accuracy and cost-effectiveness are still under investigation [12]. Despite possible future solutions, it is essential to support and collaborate in defeating TB, not only for low-income countries but also in terms of global and globalised health. Importantly, the recent COVID-19 pandemic highlighted the weakness of world health systems, and high-income countries are increasingly exposed to TB due to immigrants from regions with a high rate of TB and a high prevalence of multidrug-resistant or extensively drug-resistant TB [13]. Another important aspect is the mutual link between TB and some non-communicable diseases that represent an increasing burden worldwide. This association creates a vicious cycle, with TB increasing non-communicable disease complications and vice versa. It also makes diagnosis and management more difficult and worsens disease courses and outcomes [14]. This case contains the main characteristics of the health systems in low-income countries, including a lack of trained health workers, a lack of means in community health centres, and a lack of effective health policies in combating endemic diseases such as TB.

## 4. Conclusions

This evidence, although rising just from a case report, allows one to make some considerations. First of all, it is essential and urgent to strengthen the development and implementation of tailored health policies for all countries affected by TB, including screening protocols for migrants, in an integrated, culturally sensitive, social-determinant- driven manner. It is also crucial to enable health workers and health facilities to achieve correct and timely diagnoses. Finally, as a pharmacological approach is necessary in the treatment of TB and costs of treatments per patient are significantly high, particular attention should be paid to social determinants of TB that influence its contraction, as well as outcome, in low-resource settings.

## Figures and Tables

**Table 1:** Biochemical results performed at admission, after 2 and 5 days.

Parameter	Admission	After 2 Days	After 5 Days
WBC	**13.3 × 1** **0^3^/uL**	**15.04 × 10** **^3^** **/uL**	**14.17 × 10** **^3^** **/uL**
LYM	5.8 (43.7%)	5.5 (37%)	6.36 (44.9%)
NEUT	6.5 (48.7%)	7.9 (52.5%)	5.88 (41.4%)
RBC	4.77 × 10^6^/uL	4.62 × 10^6^/uL	4.72 × 10^6^/uL
HGB	**7.5 g/dl**	**7.4 g/dl**	**7.7 g/dl**
MCV	**57.9 fL**	**54.1 fL**	**54.2 fL**
MCH	15.7 pg	16.0 pg	16.3 pg
MCHC	**27.2 g/dL**	**29.6 g/dL**	**30.1 g/dL**
PLT	**1000 × 10** **^3^** **/uL**	**944 × 10** **^3^** **/uL**	**1030 × 10** **^3^** **/uL**
Na	133 mEq/L	NR	132.9 mEq/L
K	4.37 mEq/L	NR	5.44 mEq/L
Cl	98 mEq/L	NR	98 mEq/L
Creatinine	20.3 umol/L	NR	22.33 umol/L
ALT	16.54 U/L	NR	26 U/L
AST	31.33 U/L	NR	43 U/L
Urea	3.01 mg/dL	NR	2.47 mg/dL
Glucose	4.86 mmol/L	NR	3.5 mmol/L
ESR	51 mm/h	NR	NR
ALB	NR	NR	3.8 g/dL
Iron	NR	NR	84 mcg/dL
BPL	NR	NR	6.7 g/dL
Cholesterol	NR	NR	148 mg/dL
TBIL	NR	NR	1.1 mg/dL

Legend to Table 1. Bold are indicates the abnormal values. List of abbreviations: ALB—albumin; ALT—alanine aminotransferase; AST—aspartate aminotransferase; BPL—blood protein level; Cl—chlorine; ESR—erythrocyte sedimentation rate; HGB—haemoglobin; K—potassium; LYM—lymphocytes; MCV—mean corpuscular volume; MCH—mean corpuscular haemoglobin; MCHC—mean cell haemoglobin concentration; Na—sodium; NEUT—neutrophils; PLT—platelets; RBC—red blood cells; TBIL—total bilirubin; WBC—white blood cells.

## Data Availability

All available data are included in the manuscript.
